# Midkine and Pleiotrophin Concentrations in Amniotic Fluid in Healthy and Complicated Pregnancies

**DOI:** 10.1371/journal.pone.0153325

**Published:** 2016-04-18

**Authors:** Youn Hee Jee, Yael Lebenthal, Piya Chaemsaithong, Gai Yan, Ivana Peran, Anton Wellstein, Roberto Romero, Jeffrey Baron

**Affiliations:** 1 Section on Growth and Development, Program on Developmental Endocrinology and Genetics, Eunice Kennedy Shriver National Institute of Child Health and Human Development, National Institutes of Health, Bethesda, Maryland, United States of America; 2 Sackler Faculty of Medicine, Tel Aviv University, Tel Aviv, Israel; 3 Perinatology Research Branch, Program for Perinatal Research and Obstetrics, Division of Intramural Research, Eunice Kennedy Shriver National Institute of Child Health and Human Development, Detroit, Michigan, United States of America; 4 Department of Oncology, Georgetown University Medical Center and Lombardi Comprehensive Cancer Center, Washington DC, United States of America; Utah State University, UNITED STATES

## Abstract

**Background:**

Midkine (MDK) and pleiotrophin (PTN) are heparin-binding growth factors that, in rodents, are highly expressed in early life and decrease to undetectable levels by adulthood. The potential roles of MDK and PTN in human growth and development are not completely elucidated.

**Method and Findings:**

To delineate the role of MDK and PTN in human development, we developed high sensitivity assays to measure their concentrations in amniotic fluid (AF) at various gestational ages in both healthy and complicated pregnancies. We found that both of these growth factors could be readily measured in AF and that the concentrations were higher than most cytokines previously reported in AF.

**Conclusion:**

The concentration of MDK but not that of PTN declined with gestational age. Both MDK and PTN concentrations were found to be lower in pregnancies that were complicated by chorioamnionitis at term, raising the possibility that these growth factors might be useful as markers for infection.

## Introduction

Amniotic fluid (AF) provides an essential, complex, and dynamic milieu for the growing fetus that changes with progression of the pregnancy [1]. AF contains nutrients (carbohydrates, proteins and peptides, and lipids), growth factors, and cytokines that facilitate fetal growth. The functions and significance of individual growth factors in human AF remain incompletely understood. Various cytokines, such as interleukin (IL)-6, IL-8, IL-10, IL-11, IL-15, tumor necrosis factor (TNF)-α, transforming growth factor (TGF)-β, and vascular endothelial growth factor (VEGF), have been studied as potential markers for a variety of conditions including pre-eclampsia, intrauterine growth retardation, preterm labor, preterm premature rupture of membranes (PPROM), and intra-amniotic inflammation/infection, but the variability in concentrations of AF cytokines often results in values that overlap with those during normal pregnancies, thus limiting the clinical utility of AF cytokine measurements [2–5].

Midkine (MDK) and pleiotrophin (PTN) are two closely-related heparin-binding growth factors that are rich in both basic amino acids (arginine, lysine, and histidine) and cysteines [6–7]. The biological activities of these growth factors include promotion of growth, cell migration, tissue morphogenesis, and chemokine expression in numerous target cell types [8–9]. In rodents, these growth factors are highly expressed in early life in multiple organs and decrease to low levels by adulthood [10–14]. The roles of MDK and PTN in human growth and development *in utero* are yet to be elucidated.

Neither MDK nor PTN concentrations have been assessed in human amniotic fluid. We therefore developed high sensitivity assays to measure the concentrations of both factors in human amniotic fluid, assessing the levels at various gestational ages, in both healthy pregnancies and pregnancies with a variety of common complications.

## Materials and Methods

### Study population

**Fig 1:**
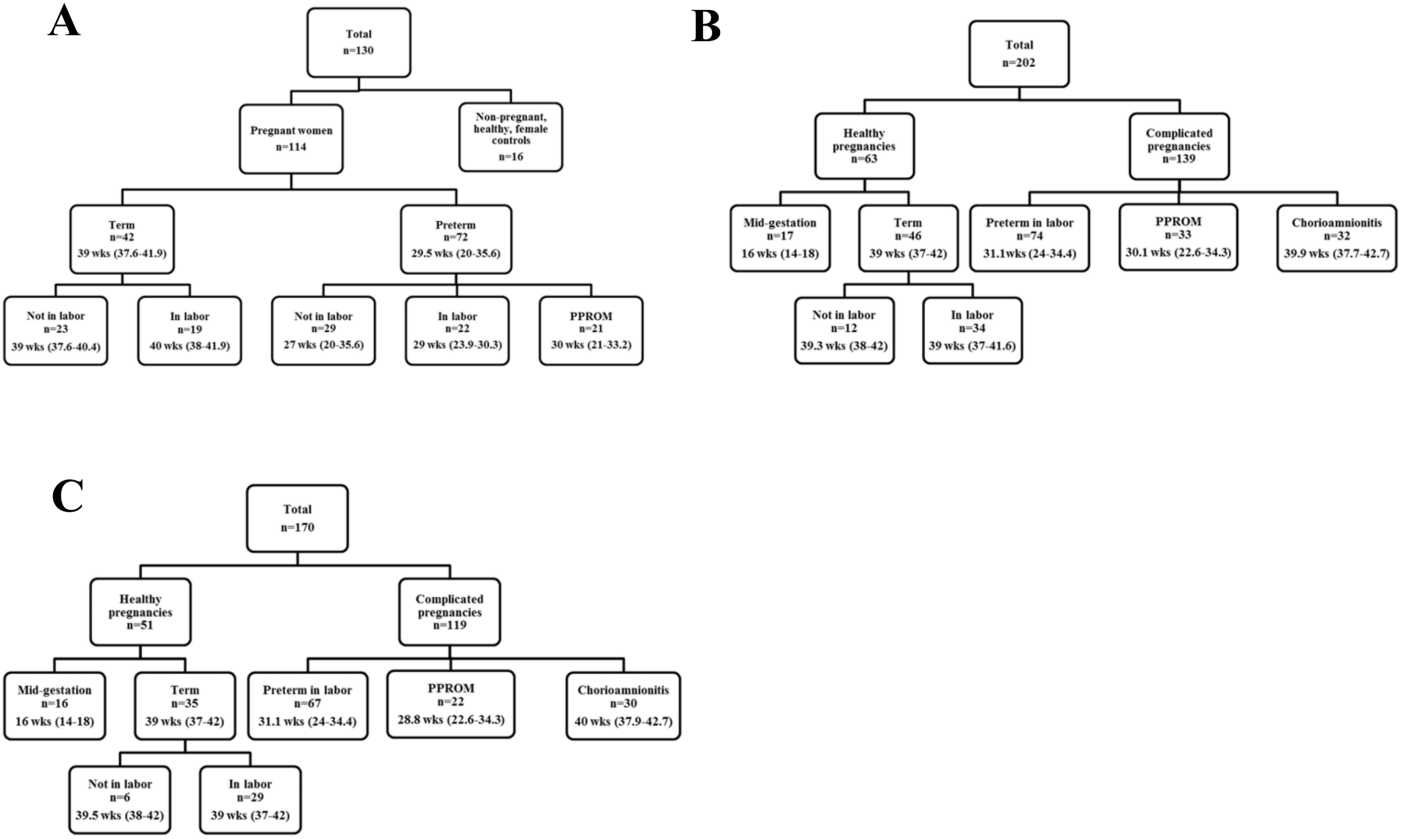
Flowchart of samples evaluated for plasma midkine (1A), amniotic fluid midkine (1B), and amniotic fluid pleiotrophin (1C). PPROM, preterm premature rupture of membranes; n, number of samples; gestational age represents the median (range) age at which sample was obtained.

Specimens were obtained from the Biologic Tissue Bank of the Perinatology Research Branch (PRB) of the *Eunice Kennedy Shriver* National Institute of Child Health and Human Development (NICHD) (ClinicalTrials.gov:NCT00340249). Plasma samples had been obtained in 1999–2001, and amniotic fluid 1990–2005. Clinical data were extracted from the associated Perinatal Database. All subjects provided written informed consent. The Institutional Review Board of the NICHD approved the use of clinical data and biological specimens. MDK was measured in maternal plasma from singleton complicated and healthy pregnancies (n = 114) and non-pregnant, healthy, age-matched controls (n = 16, Fig 1A). MDK (n = 202) and PTN (n = 170) were measured in AF from singleton pregnancies grouped according to outcome of pregnancy (Fig 1B and 1C). PTN was not measured in all samples due to insufficient sample volume.

### Biological samples and analysis

Peripheral blood was collected in a glass tube containing citrate. The blood was centrifuged at 4°C for 15 min at 3,000 g within 2 h of venipuncture. Plasma was aliquotted in plastic tubes and stored at -80°C until MDK assay. AF not required for clinical assessment was centrifuged in glass tubes for 10 min at 4°C and stored at -70°C in plastic tubes until assay. The investigator performing the assays was blinded to all clinical data.

### Clinical definitions

Gestational age (GA) was self-reported based on the last menstrual period and confirmed by ultrasound. In cases of inconsistency between reported last menses and sonographic determination of GA, ultrasonographic age was used. Term gestation was defined by gestational age 37–42 weeks. Preterm premature rupture of membranes (PPROM) was diagnosed as accumulation of AF in the vagina before 37 weeks of gestation and confirmed by a positive nitrazine test and/or positive ferning test. Clinical chorioamnionitis was diagnosed when maternal temperature exceeded 37.8°C with the presence of at least 2 of the following criteria: uterine tenderness, malodorous vaginal discharge, maternal leukocytosis (>15,000 cells/mm3), maternal tachycardia (>100 beats/min), or fetal tachycardia (>160 beats/min) [15–16].

### Midkine sandwich ELISA for plasma and amniotic fluid

MDK sandwich enzyme-linked immunosorbent assay (ELISA) was performed using a commercial kit (Biovendor, Czech Republic) with modifications as previously described [17]. In particular, poly-L-lysine was added to the biotin-labelled detection antibody solution provided with the kit to increase responsiveness of the ELISA. Prior to assay, 125 μL of plasma were diluted in 125 μL of TBSTA (TBS, 1% BSA, 0.5% Tween 20, pH 7.4) and 5 μL of AF were diluted in 245 μL of TBSTA. The rest of the procedure was identical to the procedure previously described [17]. The detection limit for plasma MDK was 8.7 pg/mL and for AF MDK was 7 pg/mL. Inter-assay CV was 3.2% for plasma and 11.7% for AF. The assay showed good parallelism in plasma (S1 Fig). The detection range of the assay was 0–0.8 ng/ml.

### Pleiotrophin sandwich ELISA for amniotic fluid

A PTN sandwich ELISA was developed in our lab. The mouse anti-pleiotrophin monoclonal antibody (3B10, produced in the lab of Dr. Anton Wellstein) was diluted to 0.5 μg/mL in PBS and 100 μL/well was incubated in a 96-well plate at 4°C overnight. The wells were washed 3 times with 250 μL per well of PBST (PBS, 0.05% Tween 20). The wells were blocked with 250 μL per well of PBS containing 3% BSA and 0.2% Tween 20 for 2 hours at 4°C. Without washing, the plate was inverted and dried by tapping vigorously against a paper towel. Subsequently, 5 μL AF were diluted in 245 μL of PBSTA (PBS, 1% BSA, 0.5% Tween 20), and 100 μL of the diluted samples were pipetted in duplicate into the wells. The plate was incubated with gentle agitation at room temperature for 2 hours and then washed 3 times with 250 uL per well of PBST. After tapping the inverted plate to remove residual fluid, a biotinylated anti-human pleiotrophin goat IgG (R & D systems, USA) were added at a concentration of 500 ng/mL in 0.9% saline containing 5.7 meq/L calcium chloride and 0.5% BSA at pH 6. The plate was incubated with gentle agitation at room temperature for an hour. Then the wells were washed 5 times with 250 μl of PBST per well. After tapping, 100 μl of streptavidin-HRP conjugate solution (Thermo Scientific, USA) were added at a concentration of 25 ng/mL in PBS to each well and the plate was incubated at room temperature for 30 minutes with gentle agitation. After washing 5 times with PBST and tapping, 100 μL of TMB (eBioscience, USA) were added to each well. The plate was covered with aluminum foil and incubated for 7 minutes at room temperature. Color development was stopped by adding 100 μl of stop solution (0.16M sulfuric acid). The absorbance of each well was measured using a microplate reader set to 450 nm (Synergy 4, BioTek, USA). The detection limit for AF PTN was 10 pg/mL. The inter-assay CV was 2.7%. The detection range of the assay was 0–1.8 ng/ml.

### Validation of AF MDK and PTN assays

**Fig 2:**
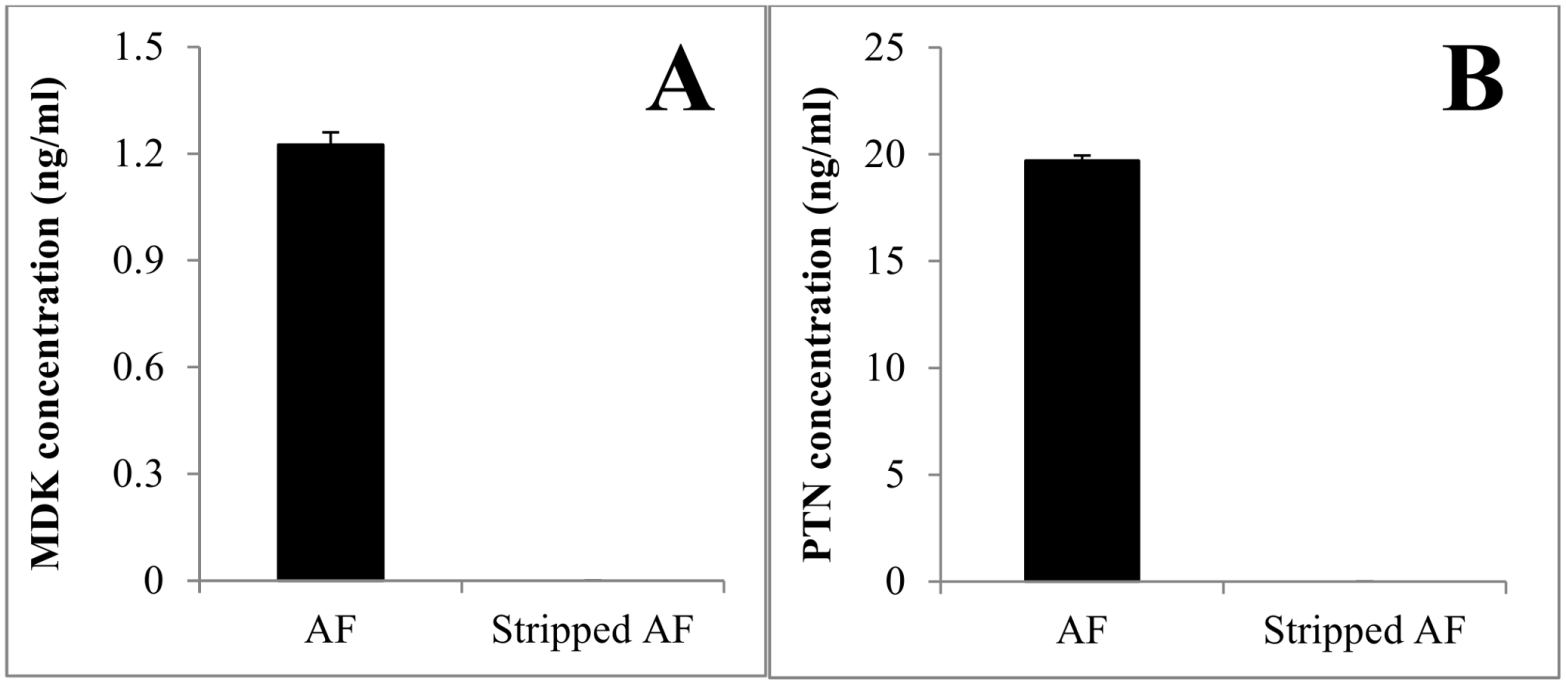
Heparin-stripping of MDK and PTN from amniotic fluid. Assay specificity was assessed by removing both MDK and PTN from AF with heparin-Sepharose beads. ELISA signals for both MDK (Panel A) and PTN (Panel B) were abolished after treatment.

To assess the specificity of the ELISAs, we took advantage of the fact that both MDK and PTN bind avidly to heparin. Both MDK and PTN were removed from AF using Heparin-Sepharose beads and this procedure essentially eliminated all ELISA signals for both MDK and PTN (Fig 2A and 2B). To further validate the MDK and PTN assays, AF was diluted serially in assay buffer prior to assay. Both the MDK and PTN assays showed good parallelism between the standard curve and serially diluted AF washout samples (S2A & S2B Fig). A 1:50 dilution of AF was then selected to perform all the MDK and PTN assays.

### Binding of MDK and PTN to collection tubes

To determine whether MDK adhered to the glass tube [18–19], blood samples from pregnant women were collected in either glass or polypropylene blood collection tubes (Becton, Dickinson and Company, Franklin Lakes, New Jersey) containing buffered sodium citrate. Plasma MDK concentrations were slightly lower (mean 17%) in the samples collected in glass tubes than in those in polypropylene tubes (S3 Fig). AF samples from the tissue bank had been centrifuged in glass tubes. To determine whether there was a loss of MDK or PTN due to adherence to glass [18–19], freshly collected AF was incubated in either polypropylene or glass tubes at room temperature for 2 hours and assayed. AF MDK concentration was slightly lower (mean 15%) after incubation in glass tubes than in polypropylene tubes, and AF PTN had a higher but still moderate loss (mean 31%) in glass tubes (S4 Fig).

### Statistical analysis

All MDK and PTN concentrations were log-transformed. Comparisons of concentrations between pairs of groups to test specific hypotheses (e.g. the effect of chorioamnionitis on growth factor levels at term) were made by *t* test. The association between gestational age and AF growth factor concentrations was examined by a general linear model that included a term for group as depicted in Fig 1B and 1C. Birth weight Z-score was calculated using the Fenton 2013 growth calculator for preterm infants [20–21]. The association between AF growth factor concentration and birth weight was assessed using a general linear model, including terms for gestational age at amniocentesis, gestational age at delivery, and group as covariates. The association between AF MDK and AF PTN was assessed by partial correlation including group as a covariate. Data are presented as mean ± SEM and were analyzed using SPSS 19 (IBM, NY). A *P* value of ≤ 0.05 was considered statistically significant.

## Results

### Midkine concentrations in plasma

**Fig 3:**
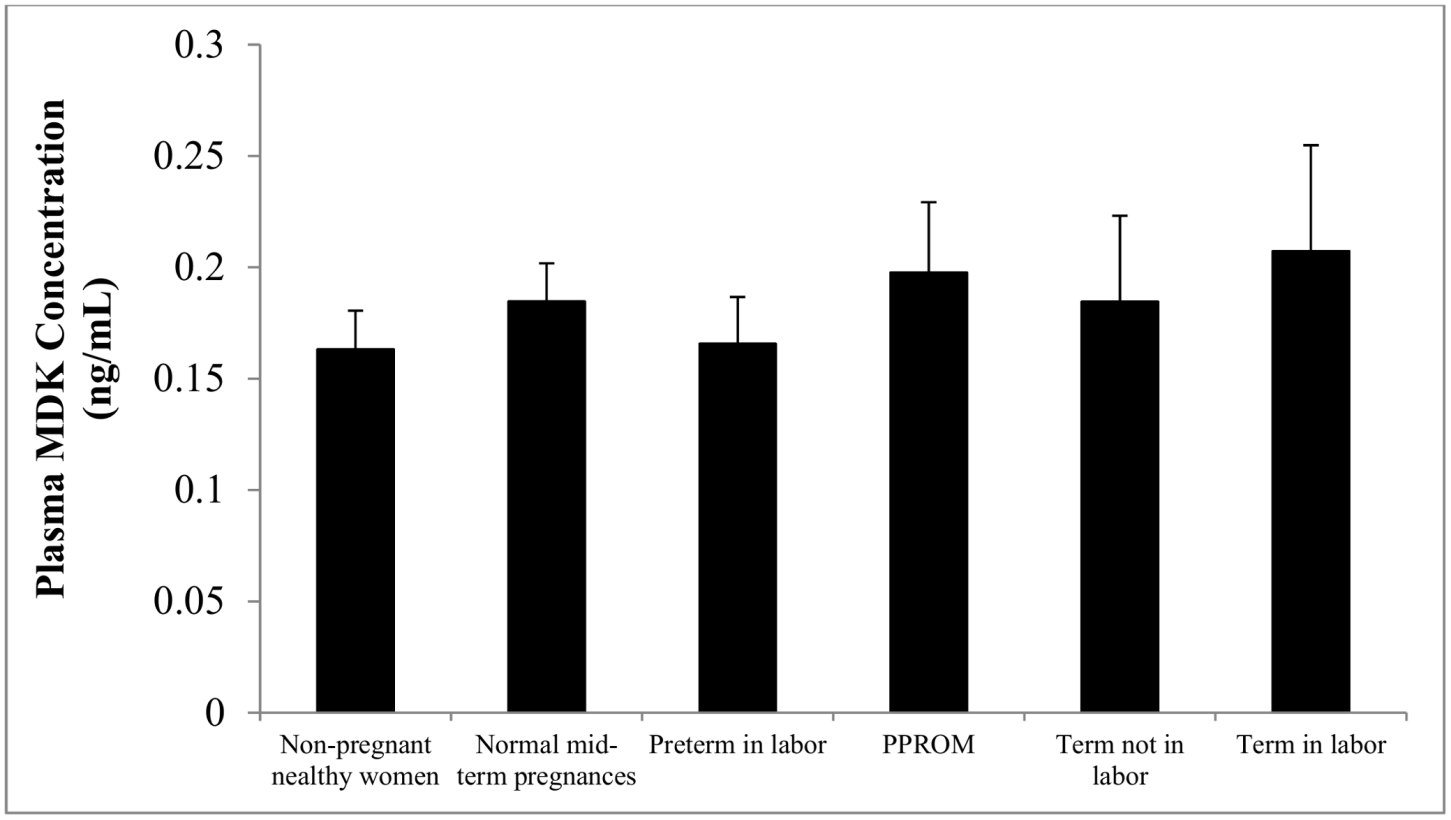
Plasma MDK concentrations during pregnancy and in non-pregnant healthy female controls. Plasma MDK concentrations were similar among non-pregnant healthy women, normal preterm pregnancies in the absence of labor, preterm in labor, PPROM, term not in labor, and term in labor. Data are presented as mean ± SEM.

The average age of the pregnant women at time of plasma sampling was similar to that of the non-pregnant healthy controls [27.6 years (18–40 years) *vs*. 25.2 years (17–37 years), *P* = 0.18]. Plasma MDK concentrations did not significantly differ between the pregnant women and non-pregnant age-matched controls (0.19 ± 0.01 ng/ml *vs*. 0.16 ± 0.02 ng/ml, *P =* 0.79). No significant differences in plasma MDK concentrations were found among non-pregnant healthy women, normal mid-term pregnancy, preterm in labor, PPROM, term without labor, and term with labor (Fig 3).

### Midkine concentrations in amniotic fluid

**Fig 4:**
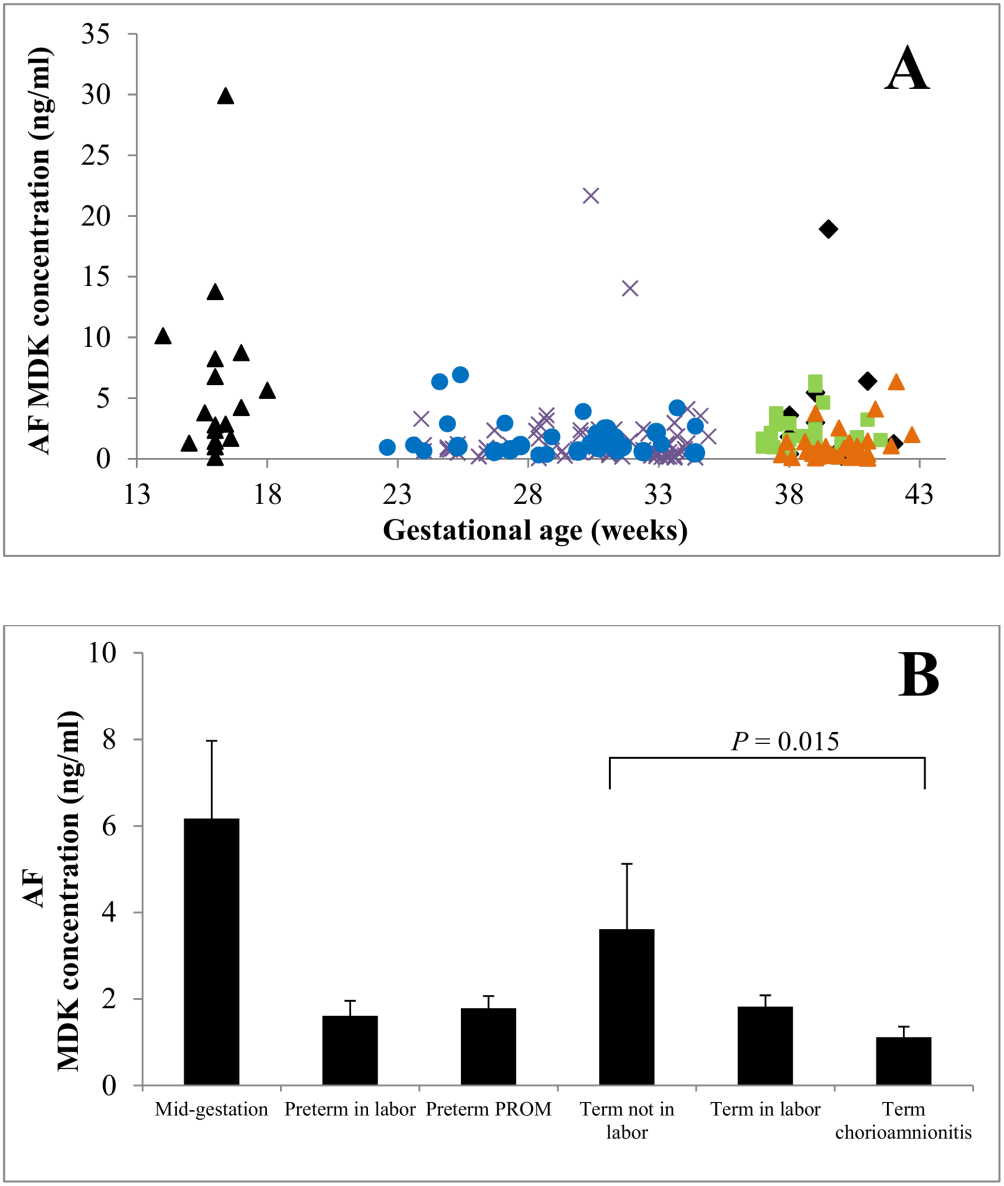
Amniotic fluid MDK concentrations. Panel A: AF MDK concentrations (n = 202) declined with gestational age. Panel B: AF MDK concentrations were not significantly different between healthy term pregnancies in the absence of labor and during labor or between pregnancies complicated by PPROM and premature labor. MDK was lower in term pregnancies complicated by chorioamnionitis than in term pregnancies without infection (P = 0.015). Data are presented as mean ± SEM. Panel A: Black triangle (mid-gestation), x (preterm labor), blue circle (premature preterm rupture of membranes), black diamond (term not in labor), green square (term in labor), orange triangle (term chorioamnionitis).

In general, MDK concentrations in AF were far higher than in maternal plasma. In healthy term pregnancies in the absence of labor, the average AF MDK concentration was 3.61 ± 1.51 ng/ml while the maternal plasma concentration was 0.18 ± 0.02 ng/ml. MDK concentrations declined with gestational age (*P* < 0.001, Fig 4A). In healthy term pregnancies, AF MDK concentrations were similar between the absence of labor and during labor (3.61 ± 1.51 ng/ml *vs*. 1.83 ± 0.26 ng/ml, *P* = 0.6, Fig 4B). Interestingly, AF MDK concentrations in term pregnancies complicated by chorioamnionitis were lower than in healthy term pregnancies in the absence of labor (1.12 ± 0.24 ng/ml *vs*. 3.61 ± 1.51, *P* = 0.015, Fig 4B). AF MDK concentrations were slightly lower in preterm pregnancies during labor than in PPROM (1.61 ± 0.35 ng/ml *vs*. 1.79 ± 0.28, *P* = 0.046, Fig 4B).

### Pleiotrophin concentrations in amniotic fluid

**Fig 5:**
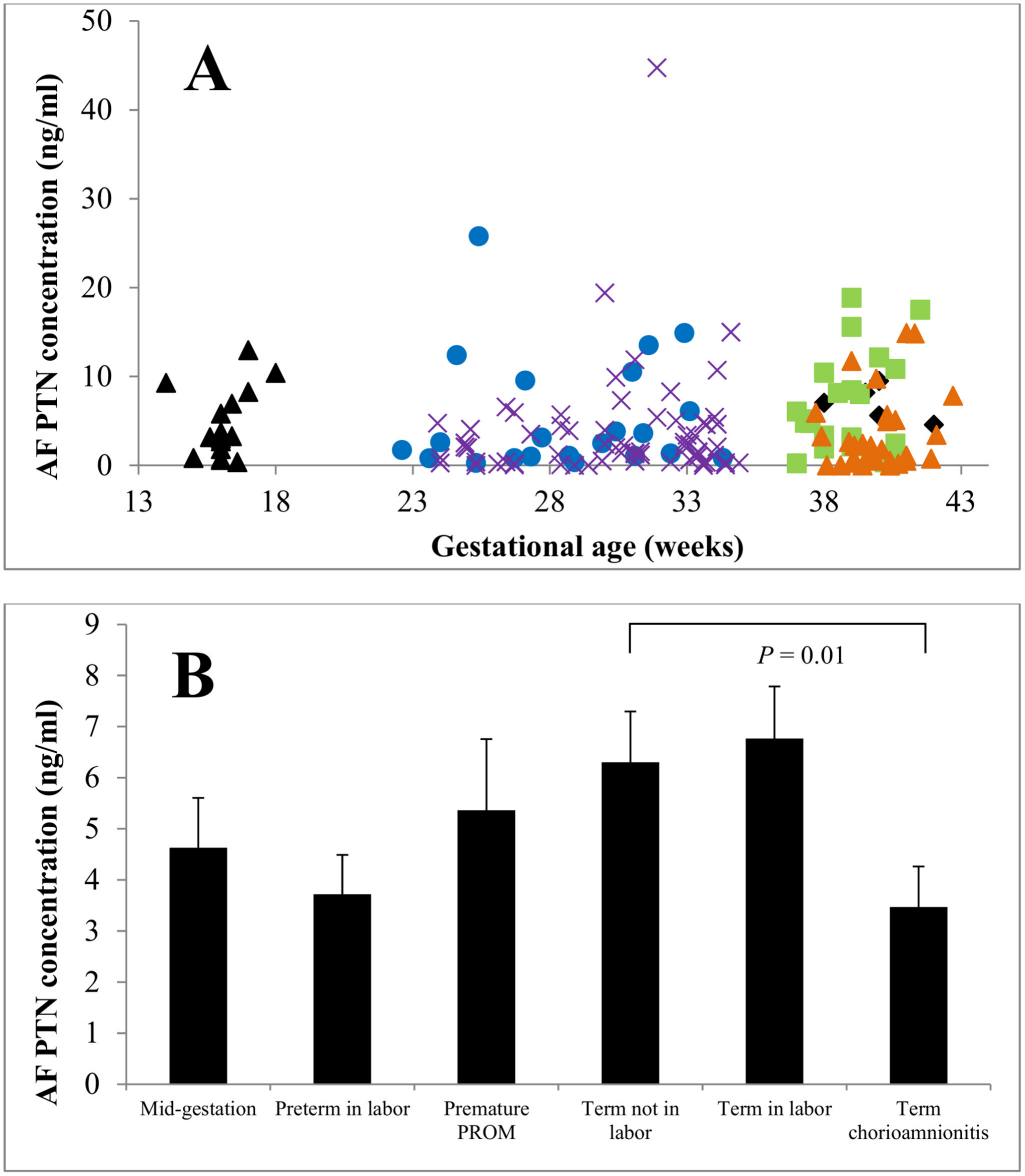
Amniotic fluid PTN concentrations. Panel A: AF PTN concentrations (n = 170) did not vary significantly with gestational age (Panel A). Panel B: AF PTN concentrations were similar between the absence and presence of labor in healthy term pregnancies, and also similar between pregnancies complicated by PPROM and by premature labor. MDK was lower in term pregnancies complicated by chorioamnionitis than in term pregnancies without infection (P = 0.01). Data are presented as mean ± SE. Panel A: Black triangle (mid-gestation), x (preterm labor), blue circle (premature preterm rupture of membranes), black diamond (term not in labor), green square (term in labor), orange triangle (term chorioamnionitis).

In healthy term pregnancies without labor, the average PTN concentration in AF was 6.3 ± 1.0 ng/ml. In contrast to AF MDK levels, AF PTN levels did not decline significantly with gestational age (*P* = 0.085, Fig 5A). AF PTN concentrations were similar in healthy term pregnancies in the absence of labor and during labor (6.30 ± 1.00 ng/ml *vs*. 6.76 ± 1.00 ng/ml, *P =* 0.534). Similar to MDK, AF PTN concentrations in term pregnancies complicated by chorioamnionitis were lower than in healthy term pregnancies in the absence of labor (3.47 ± 0.79 ng/ml *vs*. 6.30 ± 1.00 ng/ml, *P* = 0.01). AF MDK concentrations were similar between preterm pregnancies during labor and PPROM (3.72 ± 0.77 ng/ml *vs*. 5.36 ± 1.39, *P* = 0.22).

### Midkine concentrations in amniotic fluid are associated with pleiotrophin concentrations

**Fig 6:**
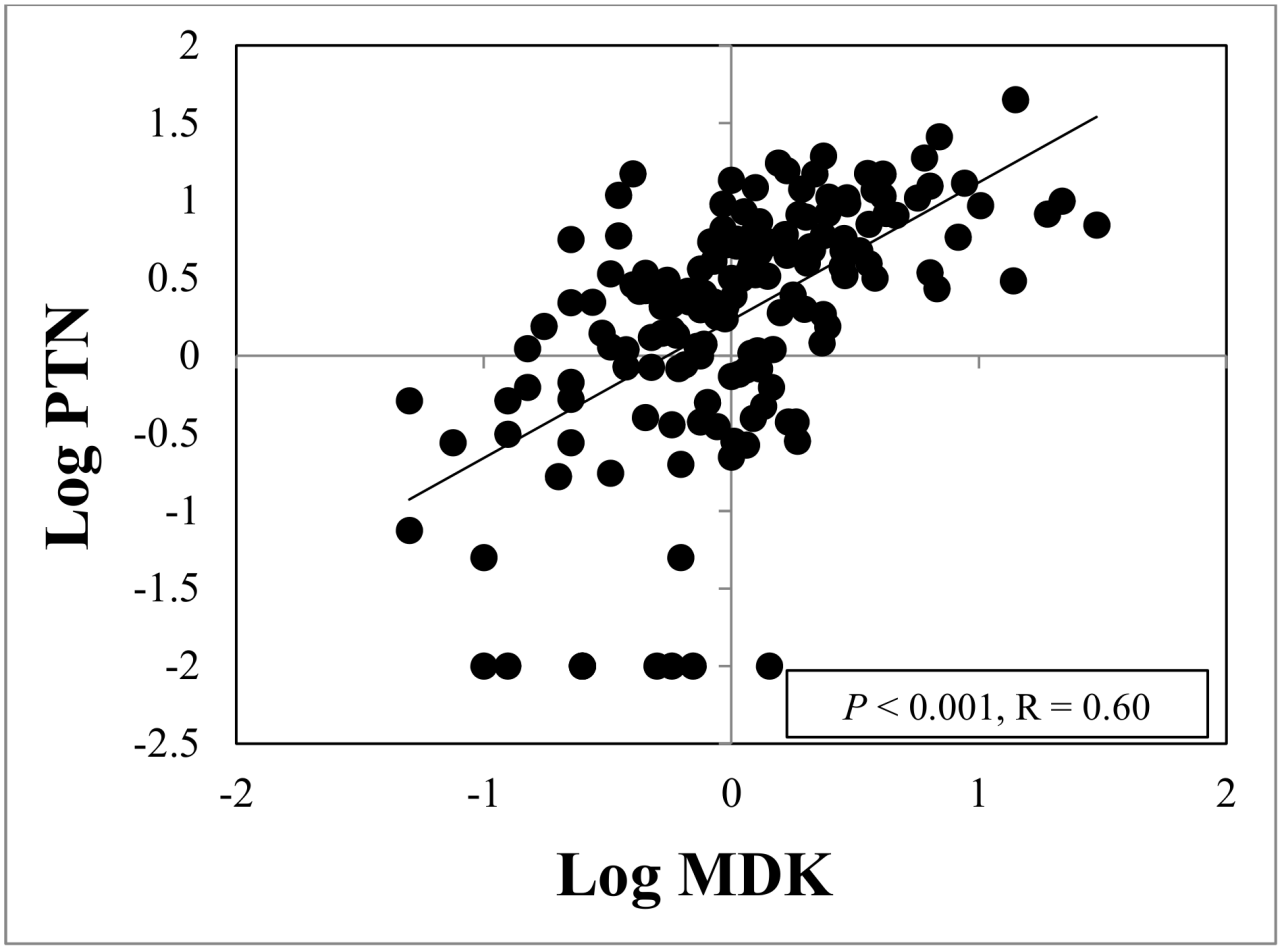
Amniotic fluid midkine and pleiotrophin concentrations correlated with each other. Midkine concentrations were positively correlated with pleiotrophin concentrations in amniotic fluid (R = 0.6, P < 0.001), using diagnostic category as a covariate.

A positive correlation between AF MDK concentrations and AF PTN concentrations was found for the study cohort (R = 0.60, *P* < 0.001, Fig 6).

## Discussion

The current study is the first to assess MDK and PTN concentrations in human AF. We found that both of these growth factors could be readily measured in AF at levels greater than those of most cytokines previously assessed in AF [2–5]. The AF concentration of MDK but not PTN declined with gestational age. Both AF MDK and PTN concentrations were lower in pregnancies complicated by chorioamnionitis than in healthy pregnancies. The presence of labor did not affect MDK or PTN levels. We also measured MDK concentrations in plasma and found that the levels did not differ significantly between pregnant and non-pregnant women. The concentration of MDK was approximately 10-fold higher in AF than in plasma.

In AF, the concentrations of MDK (approximately 2 ng/ml) and PTN (approximately 4.5 ng/ml) were substantially higher than the concentrations of other cytokines previously measured, including IL-6, IL-8, IL-10, IL-11, IL-15, TNF-α, TGF-β, and VEGF. The relative abundance of these two growth factors is consistent with previous observations that the genes encoding both factors are highly expressed in multiple embryonic tissues [11, 13–14, 22–27]. In mice, MDK was previously found to be expressed in extraembryonic membranes and present in amniotic fluid at a concentration of approximately 1 μg/ml [28]. Despite the relatively high levels of MDK in AF, its levels in maternal circulation were not elevated compared to its plasma levels in healthy non-pregnant women, suggesting that MDK does not escape from the fetal compartment in substantial quantities. In rodents, expression of both MDK and PTN [13–14] decreases with age in multiple tissues postnatally [10–12, 29–31]. We therefore anticipated that levels of these proteins might decrease with gestational age in human AF. Consistent with this expectation, MDK AF concentrations declined between mid-gestation and term. However, PTN concentrations did not change significantly with gestational age.

The MDK and PTN found in amniotic fluid may be derived from embryonic and/or extraembryonic tissues. Both of these heparin-binding growth factors are expressed at high levels in multiple embryonic tissues [12]. In addition, MDK is expressed in the placenta and extraembryonic membranes of the mouse [26, 28]. PTN expression is low in the trophoblast of most mammals but is highly expressed in the human and chimpanzee, driven by a trophoblast-specific promoter due to insertion of an endogenous retrovirus during primate evolution [27]. Therefore, in humans, amniotic fluid MDK and PTN, which we found in high concentrations, may be derived from the fetus, placenta, and/or amniotic membrane.

The overall and AF-specific functions of MDK and PTN in human development remain unclear. The developmental functions of these growth factors have been investigated in tissue culture and animal models, suggesting roles in the development of the nervous, skeletal, reproductive, and endocrine systems [32–35]. In mice, targeted genetic ablation of both genes results in reduced postnatal growth, infertility, cochlear, auditory dysfunction, and early death [36–37]. PTN may play an important role in the primate placenta. As noted above, there is evidence that insertion of a retrovirus-like element into the primate PTN gene generated an additional promoter with trophoblast specific activity. It has been suggested that the resulting high level of PTN expression may support embryo implantation into the uterus and drive invasion of the trophoblast into the uterine muscle [38–40].

Interestingly, both MDK and PTN concentrations were lower in term samples from pregnancies complicated by chorioamnionitis than in those from healthy pregnancies, suggesting that infection either decreases the expression or accelerates the degradation of these growth factors. This observation also raises the possibility that measuring MDK and PTN in AF might serve as an adjunctive diagnostic tool to determine the presence of infection. Whether the decreased AF MDK and PTN exert functional effects on the fetus is unknown.

Although this study demonstrates the presence of MDK and PTN in human AF at higher concentrations than other cytokines, the subgroup analysis comparing different gestational ages and different pregnancy complications was limited by the nature of the sample set. For ethical reasons, AF can only be obtained when amniocentesis is clinically indicated. Thus, for example, at 22 to 35 weeks of gestation, we could obtain samples from women with PPROM but not from those with healthy pregnancies. For similar practical reasons, the study used cross-sectional sampling rather than longitudinal sampling from each subject, limiting our ability to analyze the natural history of how the levels of these heparin-binding growth factors are regulated through the course of normal pregnancy. In addition, the samples for this study were obtained from an established tissue bank, which further limited the number, diagnoses, and gestational age of subjects available. For example, our sample population did not include pregnancies complicated by intrauterine growth retardation or overgrowth, a group of considerable interest given the requirement for these two growth factors for normal growth in mice [36]. Furthermore, because the plasma and AF samples were not taken from the same subjects for MDK measurement, the correlation between the two sets of measurement could not be determined. Plasma PTN concentrations were not determined because the assay developed in our lab showed strong interference from matrix effects and consequently poor parallelism. To build upon the findings of this pilot study and to address its limitations, further studies would be of interest, exploring the changes of these growth factor levels with gestational age, their relationships to fetal growth, and their alterations during pregnancy complications that were not addressed in this preliminary study.

## Conclusions

In conclusion, we have successfully developed methods to measure both MDK and PTN concentrations in AF and found that both growth factors were present at higher levels than most other cytokines previously measured in AF. For MDK, the levels in AF were also substantially higher than in maternal plasma. MDK but not PTN concentrations declined with gestational age. Both MDK and PTN levels were found to be lower in pregnancies complicated by chorioamnionitis, suggesting that these growth factors might be used clinically as markers for infection. Further investigation is needed to determine the roles of these growth factors in human gestation, particularly whether or not MDK or PTN regulate human growth *in utero* and whether abnormalities involving these growth factors might contribute to intrauterine growth retardation.

## Supporting Information

S1 Fig Parallelism between the standard curve and serially diluted samples of the MDK ELISA. A plasma sample was diluted 2, 10, 50, and 100 fold into assay buffer (TBSTA) and then assayed for MDK. Black bars, measured MDK concentrations; patterned gray bars, measured value multiplied by the dilution factor. Data are presented as mean ± SEM.
(DOCX)

S2 Fig A & B. Parallelism between the standard curve and serially diluted amniotic fluid washout samples. An amniotic fluid sample was diluted 100, 200, and 400 fold into assay buffer for MDK measurement (Panel A) and 25, 50, and 100 fold for PTN measurement (Panel B). The assays showed good parallelism between the standard curve and serially diluted AF washout samples. Black bars, measured MDK/PTN concentrations; patterned gray bars, measured value multiplied by the dilution factor. Data are presented as mean ± SEM.
(DOCX)

S3 Fig Comparison of MDK concentrations in plasma collected in polypropylene vs. glass tubes. Plasma in the tissue bank had been collected in glass citrate tubes, centrifuged promptly, and stored at -80°C in polypropylene tubes. To determine whether MDK adhered to the glass tubes (Hando et al., 2008), freshly obtained blood samples (n = 5) from pregnant women were collected in either glass or polypropylene blood collection tubes (Becton, Dickinson and Company, Franklin Lakes, New Jersey) containing sodium citrate, incubated for 2 hours at room temperature, and centrifuged. The plasma was then transferred to polypropylene storage tubes and frozen at -80°C until subsequent analysis of MDK. Plasma MDK concentrations (mean ± SEM of replicates) were slightly higher in polypropylene (black bars) than in glass collection tubes (patterned gray bars).
(DOCX)

S4 Fig A & B. Comparison of MDK and PTN concentrations in AF collected in polypropylene vs. glass tubes. AF in the tissue bank had been centrifuged in glass tubes. To determine whether MDK or PTN adheres to the glass (Hando et al., 2008), freshly collected AF (n = 5) was placed in either polypropylene or glass tubes, stored at room temperature for 2 hours, aliquotted into polypropylene tubes, frozen at -80 C, and later assayed for MDK and PTN. AF MDK (Panel A) and PTN (Panel B) concentrations (mean ± SEM of replicates) were slightly higher in polypropylene (black bars) than in glass collection tubes (patterned gray bars).
(DOCX)

## References

[1] Underwood MA, Gilbert WM, Sherman MP. 2005 Amniotic fluid: not just fetal urine anymore. J Perinatol 25:341–348. . 15861199 10.1038/sj.jp.7211290

[2] Heikkinen J, Möttönen M, Pulkki K, Lassila O, Alanen A. 2001 Cytokine levels in midtrimester amniotic fluid in normal pregnancy and in the prediction of pre-eclampsia. Scand J Immunol 53:310–314. . 11251890 10.1046/j.1365-3083.2001.00872.x

[3] Ozgu-Erdinc AS, Cavkaytar S, Aktulay A, Buyukkagnici U, Erkaya S, Danisman N. 2014 Mid-trimester maternal serum and amniotic fluid biomarkers for the prediction of preterm delivery and intrauterine growth retardation. J Obstet Gynaecol Res. 40:1540–1546. 10.1111/jog.12371 24888913

[4] Hong SN, Joo BS, Chun S, Kim A, Kim HY. 2015 Prediction of preterm delivery using levels of vascular endothelial growth factor and leptin in amniotic fluid from the second trimester. Arch Gynecol Obstet 291:265–271. 10.1007/s00404-014-3439-6 25266872

[5] Chaemsaithong P, Romero R, Korzeniewski SJ, Martinez-Varea A, Dong Z, Yoon BH, et al 2015 A point of care test for interleukin-6 in amniotic fluid in preterm prelabor rupture of membranes: a step toward the early treatment of acute intra-amniotic inflammation/infection. J Matern Fetal Neonatal Med 23:1–8. . 25758620 10.3109/14767058.2015.1006621 PMC5703063

[6] Erguven M, Muramatsu T, Bilir A. 2012 Midkine: From Embryogenesis to Pathogenesis and Therapy. Dordrecht, the Netherlands: Springer.

[7] Muramatsu T. 2014 Structure and function of midkine as the basis of its pharmacological effects. Br J Pharmacol 171:814–826. 10.1111/bph.12353 . 23992440 PMC3925020

[8] Kadomatsu K, Kishida S, Tsubota S. 2013 The heparin-binding growth factor midkine: the biological activities and candidate receptors. J Biochem 153:511–521. 10.1093/jb/mvt035 23625998

[9] Jones DR. 2014 Measuring midkine: the utility of midkine as a biomarker in cancer and other diseases. Br J Pharmacol 171:2925–2939. 10.1111/bph.12601 24460734 PMC4055197

[10] Matsubara S, Tomomura M, Kadomatsu K, Muramatsu T. 1990 Structure of a retinoic acid-responsive gene, MK, which is transiently activated during the differentiation of embryonal carcinoma cells and the mid-gestation period of mouse embryogenesis. J Biol Chem 265:9441–9443. . 2345177

[11] Kadomatsu K, Huang RP, Suganuma T, Murata F, Muramatsu T. 1990 A retinoic acid responsive gene MK found in the teratocarcinoma system is expressed in spatially and temporally controlled manner during mouse embryogenesis. J Cell Biol 110:607–616. . 1689730 10.1083/jcb.110.3.607 PMC2116029

[12] Mitsiadis TA, Salmivirta M, Muramatsu T, Muramatsu H, Rauvala H, Lehtonen E et al 1995 Expression of the heparin-binding cytokines, midkine (MK) and HB-GAM (pleiotrophin) is associated with epithelial-mesenchymal interactions during fetal development and organogenesis. Development 121:37–51. . 7867507 10.1242/dev.121.1.37

[13] Finkielstain GP, Forcinito P, Lui JC, Barnes KM, Marino R, Makaroun S et al 2009 An extensive genetic program occurring during postnatal growth in multiple tissues. Endocrinology 150:1791–1800. 10.1210/en.2008-0868 19036884 PMC2659288

[14] Lui JC, Forcinito P, Chang M, Chen W, Barnes KM, Baron J. 2010 Coordinated postnatal down-regulation of multiple growth-promoting genes: evidence for a genetic program limiting organ growth. FASEB J 24:3083–3092. 10.1096/fj.09-152835 20371622 PMC2909290

[15] Gibbs RS, Dinsmoor MJ, Newton ER, Ramamurthy RS. 1988 A randomized trial of intrapartum versus immediate postpartum treatment of women with intra-amniotic infection. Obstet Gynecol 72:823–828. . 3186087 10.1097/00006250-198812000-00001

[16] Redline RW, Heller D, Keating S, Kingdom J. 2005 Placental diagnostic criteria and clinical correlation-a workshop report. Placenta 26 Suppl A:S114–117. . 15837060 10.1016/j.placenta.2005.02.009

[17] Jee YH, Celi FC, Sampson M, Sacks DB, Remaley AT, Kebebew E et al 2014 Midkine concentrations in fine-needle aspiration of benign and malignant thyroid nodules. Clin Endocrinol. In Press. . 25411136 10.1111/cen.12676 PMC5532878

[18] Goebel-Stengel M, Stengel A, Taché Y, Reeve JR Jr. 2011 The importance of using the optimal plasticware and glassware in studies involving peptides. Anal Biochem 414:38–46. 10.1016/j.ab.2011.02.009 21315060 PMC3290000

[19] Hando A, Takesima S, Takahama M, Itoh S, Yokoo T, Kasai M, et al 2008 [Pre-analytical problems on assay conditions for blood midkine]. Rinsho Byori 56:221–227. Japanese. . 18411806

[20] Fenton TR, Kim JH. 2013 A systematic review and meta-analysis to revise the Fenton growth chart for preterm infants. BMC Pediatr 13:59 10.1186/1471-2431-13-59 23601190 PMC3637477

[21] Fenton 2013 Growth Calculator for Preterm Infants PediTools [Online] Available: http://www.peditools.org/fenton2013/index.php. Accessed: April 2015.

[22] Nakamoto M, Matsubara S, Miyauchi T, Obama H, Ozawa M, Muramatsu T. 1992 A new family of heparin binding growth/differentiation factors: differential expression of the midkine (MK) and HB-GAM genes during mouse development. J Biochem 112: 346–349. . 1343086 10.1093/oxfordjournals.jbchem.a123903

[23] Uehara K, Matsubara S, Kadomatsu K, Tsutsui J, Muramatsu T. 1992 Genomic structure of human midkine (MK), a retinoic acid-responsive growth/differentiation factor. J Biochem 111: 563–567. . 1639750 10.1093/oxfordjournals.jbchem.a123797

[24] Muramatsu H, Shirahama H, Yonezawa S, Maruta H, Muramatsu T. 1993 Midkine, a retinoic acid-inducible growth/differentiation factor: immunochemical evidence for the function and distribution. Dev Biol 159: 392–402. 8405666 10.1006/dbio.1993.1250

[25] Satoh J, Muramatsu H, Moretto G, Muramatsu T, Chang HJ, Kim ST et al 1993 Midkine that promotes survival of fetal human neurons is produced by fetal human astrocytes in culture. Brain Res Dev Brain Res 75: 201–205. . 8261612 10.1016/0165-3806(93)90024-5

[26] Fan QW, Muramatsu T, Kadomatsu K. 2000 Distinct expression of midkine and pleiotrophin in the spinal cord and placental tissues during early mouse development. Dev Growth Differ 42:113–119. 10830434 10.1046/j.1440-169x.2000.00497.x

[27] Ball M, Carmody M, Wynne F, Dockery P, Aigner A, Cameron I et al 2009 Expression of pleiotrophin and its receptors in human placenta suggests roles in trophoblast life cycle and angiogenesis. Placenta 30:649–53. 10.1016/j.placenta.2009.05.001 19481257

[28] Obama H, Tsutsui J, Ozawa M, Yoshida H, Yoshida Y, Osame M et al 1995 Midkine (MK) expression in extraembryonic tissues, amniotic fluid, and cerebrospinal fluid during mouse embryogenesis. J Biochem 118:88–93. . 8537330 10.1093/oxfordjournals.jbchem.a124896

[29] Bloch B, Normand E, Kovesdi I, Böhlen P. 1992 Expression of the HBNF (heparin-binding neurite-promoting factor) gene in the brain of fetal, neonatal and adult rat: an in situ hybridization study. Brain Res Dev Brain Res. 70: 267–278. 1477961 10.1016/0165-3806(92)90206-c

[30] Vanderwinden JM, Mailleux P, Schiffmann SN, Vanderhaeghen JJ. 1992 Cellular distribution of the new growth factor pleiotrophin (HB-GAM) mRNA in developing and adult rat tissues. Anat Embryol 186:387–406. . 1416088 10.1007/BF00185989

[31] Qi M, Ikematsu S, Ichihara-Tanaka K, Sakuma S, Muramatsu T, Kadomatsu K. 2000 Midkine rescues Wilms' tumor cells from cisplatin-induced apoptosis: regulation of Bcl-2 expression by Midkine. J Biochem 127: 269–277. 10731694 10.1093/oxfordjournals.jbchem.a022604

[32] Ishimoto H, Muench MO, Higuchi T, Minegishi K, Tanaka M, Yoshimura Y et al 2006 Midkine, a heparin-binding growth factor, selectively stimulates proliferation of definitive zone cells of the human fetal adrenal gland. J Clin Endocrinol Metab 91:4050–4056. . 16895951 10.1210/jc.2006-1139

[33] Winkler C, Yao S. 2014 The midkine family of growth factors: diverse roles in nervous system formation and maintenance. Br J Pharmacol.171(4):905–12. 10.1111/bph.12462 24125182 PMC3925029

[34] Liedert A, Schinke T, Ignatius A, Amling M. 2014 The role of midkine in skeletal remodelling. Br J Pharmacol. 171(4):870–8. 10.1111/bph.12412 24102259 PMC3925025

[35] Muramatsu T. 2010 Midkine, a heparin-binding cytokine with multiple roles in development, repair and diseases. Proc Jpn Acad Ser B Phys Biol Sci. 2010;86(4):410–25. 20431264 10.2183/pjab.86.410 PMC3417803

[36] Muramatsu H, Zou P, Kurosawa N, Ichihara-Tanaka K, Maruyama K, Inoh K et al 2006 Female infertility in mice deficient in midkine and pleiotrophin, which form a distinct family of growth factors. Genes Cells 11:1405–1417. . 17121547 10.1111/j.1365-2443.2006.01028.x

[37] Zou P, Muramatsu H, Sone M, Hayashi H, Nakashima T, Muramatsu T. 2006 Mice doubly deficient in the midkine and pleiotrophin genes exhibit deficits in the expression of beta-tectorin gene and in auditory response. Lab Invest 86:645–653. . 16619002 10.1038/labinvest.3700428

[38] Schulte AM, Malerczyk C, Cabal-Manzano R, Gajarsa JJ, List HJ, Riegel AT et al Influence of the human endogenous retrovirus-like element HERV-E.PTN on the expression of growth factor pleiotrophin: a critical role of a retroviral Sp1-binding site. Oncogene (2000) vol. 19 (35) pp. 3988–98 10962555 10.1038/sj.onc.1203742

[39] Schulte AM, Lai S, Kurtz A, Czubayko F, Riegel AT, Wellstein A. Human trophoblast and choriocarcinoma expression of the growth factor pleiotrophin attributable to germ-line insertion of an endogenous retrovirus. Proc Natl Acad Sci USA (1996) vol. 93 (25) pp. 14759–64 8962128 10.1073/pnas.93.25.14759 PMC26209

[40] Schulte AM, Wellstein A. Structure and phylogenetic analysis of an endogenous retrovirus inserted into the human growth factor gene pleiotrophin. J Virol (1998) vol. 72 (7) pp. 6065–72 9621070 10.1128/jvi.72.7.6065-6072.1998 PMC110412

