# Supplementary material for: Midkine and Pleiotrophin Concentrations in Amniotic Fluid in Healthy and Complicated Pregnancies
Source: PLoS One. 2016 Apr 18;11(4):e0153325. doi: 10.1371/journal.pone.0153325 (PMC4835047; doi:10.1371/journal.pone.0153325)
Supplement: S1 Fig — A plasma sample was diluted 2, 10, 50, and 100 fold into assay buffer (TBSTA) and then assayed for MDK. Black bars, measured MDK concentrations; patterned gray bars, measured value multiplied by the dilution factor. Data are presented as mean ± SEM. (DOCX) [file pone.0153325.s001.docx]

Supplemental Materials

S1 Fig.
