# Supplementary material for: Midkine and Pleiotrophin Concentrations in Amniotic Fluid in Healthy and Complicated Pregnancies
Source: PLoS One. 2016 Apr 18;11(4):e0153325. doi: 10.1371/journal.pone.0153325 (PMC4835047; doi:10.1371/journal.pone.0153325)
Supplement: S2 Fig — An amniotic fluid sample was diluted 100, 200, and 400 fold into assay buffer for MDK measurement (Panel A) and 25, 50, and 100 fold for PTN measurement (Panel B). The assays showed good parallelism between the standard curve and serially diluted AF washout samples. Black bars, measured MDK/PTN concentrations; patterned gray bars, measured value multiplied by the dilution factor. Data are presented as mean ± SEM. (DOCX) [file pone.0153325.s002.docx]

Supplemental Materials

S2A & 2B Fig.
