# Supplementary material for: Midkine and Pleiotrophin Concentrations in Amniotic Fluid in Healthy and Complicated Pregnancies
Source: PLoS One. 2016 Apr 18;11(4):e0153325. doi: 10.1371/journal.pone.0153325 (PMC4835047; doi:10.1371/journal.pone.0153325)
Supplement: S3 Fig — Plasma in the tissue bank had been collected in glass citrate tubes, centrifuged promptly, and stored at -80°C in polypropylene tubes. To determine whether MDK adhered to the glass tubes (Hando et al., 2008), freshly obtained blood samples (n = 5) from pregnant women were collected in either glass or polypropylene blood collection tubes (Becton, Dickinson and Company, Franklin Lakes, New Jersey) containing sodium citrate, incubated for 2 hours at room temperature, and centrifuged. The plasma was then transferred to polypropylene storage tubes and frozen at -80°C until subsequent analysis of MDK. Plasma MDK concentrations (mean ± SEM of replicates) were slightly higher in polypropylene (black bars) than in glass collection tubes (patterned gray bars). (DOCX) [file pone.0153325.s003.docx]

Supplemental Materials

S3 Fig.
