# Supplementary material for: Midkine and Pleiotrophin Concentrations in Amniotic Fluid in Healthy and Complicated Pregnancies
Source: PLoS One. 2016 Apr 18;11(4):e0153325. doi: 10.1371/journal.pone.0153325 (PMC4835047; doi:10.1371/journal.pone.0153325)
Supplement: S4 Fig — AF in the tissue bank had been centrifuged in glass tubes. To determine whether MDK or PTN adheres to the glass (Hando et al., 2008), freshly collected AF (n = 5) was placed in either polypropylene or glass tubes, stored at room temperature for 2 hours, aliquotted into polypropylene tubes, frozen at -80 C, and later assayed for MDK and PTN. AF MDK (Panel A) and PTN (Panel B) concentrations (mean ± SEM of replicates) were slightly higher in polypropylene (black bars) than in glass collection tubes (patterned gray bars). (DOCX) [file pone.0153325.s004.docx]

Supplemental Materials

S4A & 4B Fig.
